# Navigating the crossroads: cardiometabolic risks in cancer survivorship – a comprehensive review

**DOI:** 10.1186/s40959-024-00240-2

**Published:** 2024-06-15

**Authors:** Arif Albulushi, Aisha Al Balushi, Muhhamed Shahzad, Ismail Al Bulushi, Hatim Al Lawati

**Affiliations:** 1 Department of Adult Cardiology, National Heart Center, The Royal Hospital, Muscat, Oman; 2 National Hyperbaric Medicine Centre, The Royal Hospital, Muscat, Oman

**Keywords:** Cardiometabolic Risks, Cancer Survivorship, Oncologic Treatment, Cardiovascular Disease, Multidisciplinary Management

## Abstract

The landscape of cancer survivorship is increasingly populated by individuals facing a spectrum of cardiometabolic risks, attributed to both their oncological history and treatment regimens. This manuscript synthesizes findings from various studies, highlighting the prevalence of traditional risk factors—hypertension, dyslipidemia, diabetes—as well as emergent concerns like obesity and metabolic syndrome among survivors. The impact of demographic variables, specific cancer types, and treatment modalities on cardiometabolic health is explored. Through a lens of multidisciplinary management and future research directives, we advocate for an integrative approach to cardiometabolic health in cancer survivors, aiming to ensure their victory over cancer extends into long-term well-being.

**Graphical Abstract:**

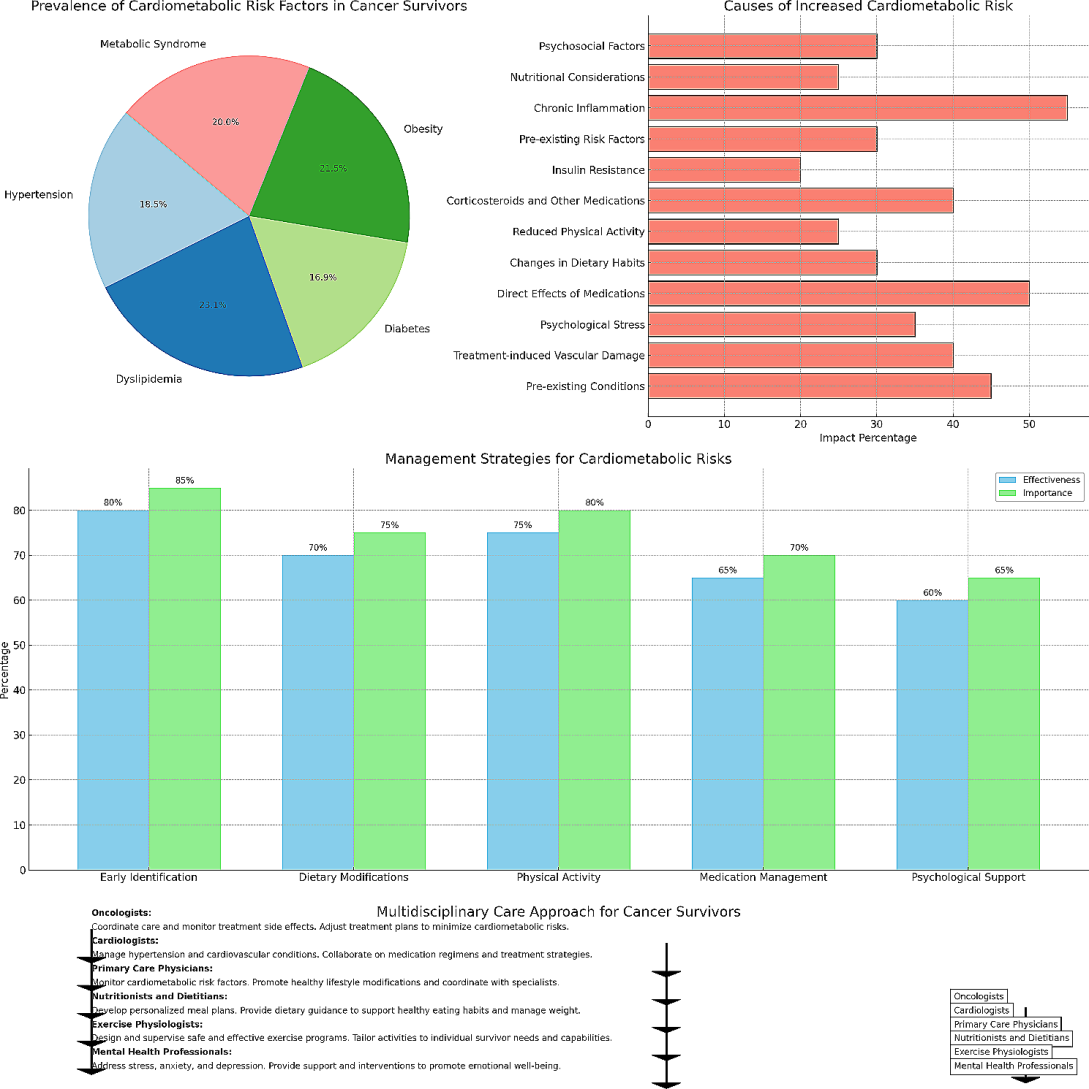

## Introduction

As we witness a rise in cancer survival rates, a parallel emergence of complex health dilemmas confronts the medical community [1]. The narrative of cancer survivorship is increasingly shadowed by the risk of cardiometabolic diseases, a multifaceted issue that intertwines with the effects of cancer therapies and the survivor’s lifestyle and genetic backdrop [2]. The intersection of oncology and cardiometabolic health represents a critical juncture in post-cancer care, one that requires an incisive understanding of its scope, impact, and the nuances of its management [3].

The concept of survivorship has evolved, transitioning from the singular goal of cancer remission to the broader vision of preserving the quality of life in the aftermath of treatment [4]. This expanded perspective necessitates attention to cardiometabolic health, given the evidence delineating a heightened risk profile for conditions such as hypertension, dyslipidemia, diabetes, obesity, and metabolic syndrome among those who have triumphed over cancer [5, 6]. This risk profile not only reflects the direct effects of oncologic treatments but also encompasses the collateral damage from lifestyle disruptions and pre-existing conditions magnified by the cancer journey [7].

The cardiometabolic repercussions for cancer survivors may be immediate or insidious, presenting as acute manifestations or as a slow accrual of risk that precipitates morbidity and threatens longevity [8]. Studies have shown that uncontrolled cardiometabolic risk factors significantly increase the likelihood of future cardiovascular events and impact overall survival rates in cancer survivors [9]. The prevalence of these conditions among survivors often mirrors or exceeds that within the general population, signaling an urgent need for integrative care strategies that are responsive to the distinct epidemiological patterns of this group [10]​​.

Furthermore, the cardiometabolic risks in cancer survivors are not uniform but are influenced by a confluence of factors including age, gender, ethnicity, and the length of survivorship [11]. Such demographic variations, alongside the type of cancer and the specific modalities of treatment received, such as radiation and chemotherapy, contribute to the heterogeneity of risk [12, 13]. The intersectional impact of these factors underscores the imperative for personalized care that addresses the full spectrum of survivor health needs, extending beyond the oncologic to embrace cardiometabolic well-being.

In this expanded framework of survivorship, the medical community is called to an in-depth exploration of the cardiometabolic risks that beset this vulnerable population. It is a call to not only chart the contours of these risks but to actively engage in mitigating their impact through evidence-based interventions and vigilant long-term care. The following sections will elucidate the prevalence and etiologies of these cardiometabolic risks, the challenges and triumphs in their management, and the uncharted frontiers in survivorship research, all aimed at safeguarding the heart health of those who have already endured the ordeal of cancer.

## Cardiometabolic comorbidities: a closer examination

### Prevalence and etiologies

#### Hypertension

The specter of hypertension looms large over cancer survivors, with its prevalence soaring between 30% and 60% compared to the general population [14]. This alarming rise stems from a confluence of factors:

- Pre-existing conditions: Many cancer survivors may have had undiagnosed hypertension before their diagnosis, which becomes more evident or worsens during treatment [15].
- Treatment-induced vascular damage: Certain cancer therapies, such as radiation therapy and chemotherapy, can directly damage blood vessels, leading to hypertension [16].
- Psychological stress: The chronic stress associated with cancer diagnosis and treatment can contribute to elevated blood pressure [17].

#### Dyslipidemia

Abnormalities in lipid profiles, characterized by elevated LDL cholesterol and triglycerides and/or reduced HDL cholesterol, plague around 40–80% of cancer survivors [14]. These alterations can be attributed to:

- Direct effects of medications: Some cancer medications, such as corticosteroids, aromatase inhibitors, and androgen deprivation therapy, can disrupt lipid metabolism and promote the buildup of harmful cholesterol [18].
- Changes in dietary habits: During treatment, cancer survivors may experience changes in appetite or taste, leading to a decreased intake of fruits, vegetables, and whole grains, and an increased consumption of processed foods, all of which can contribute to dyslipidemia [19].
- Reduced physical activity: Fatigue and treatment side effects can lead to decreased physical activity, which can contribute to a sluggish metabolism and dyslipidemia [20].

#### Diabetes

The risk of developing diabetes mellitus after a cancer diagnosis shoots up by two to threefold compared to the general population [14]. Several factors contribute to this increased risk:

- Corticosteroids and other medications: These medications can directly impair insulin sensitivity and glucose metabolism, paving the way for diabetes [21].
- Insulin resistance: Cancer itself can trigger insulin resistance, making it more difficult for the body to use insulin effectively [22].
- Pre-existing risk factors: Obesity and sedentary lifestyle, common among cancer survivors, further amplify the susceptibility to diabetes [23].

#### Emerging concerns

Beyond the traditional trio of hypertension, dyslipidemia, and diabetes, newer threats lurk in the shadows for cancer survivors:

- Inflammation: This plays a critical role in the pathogenesis of cardiovascular disease, especially in cancer survivors. Chronic inflammation, often exacerbated by obesity and metabolic syndrome, contributes to the progression of atherosclerosis and other cardiovascular conditions [24].
- Obesity: Affecting 25–60% of cancer survivors, obesity not only poses its own health risks but also serves as a breeding ground for other cardiometabolic complications [23].
- Metabolic syndrome: This constellation of risk factors, including central obesity, hypertension, dyslipidemia, and insulin resistance, afflicts up to 40% of cancer survivors, significantly amplifying their risk for cardiovascular disease [25].
- Nutritional Considerations: Provide detailed guidance on dietary choices that can mitigate cardiometabolic risks.
- Psychosocial Factors: Explore the role of stress, anxiety, and depression in the development and management of these comorbidities.

### Management strategies

Effectively managing the intricate tapestry of cardiometabolic risks in cancer survivors necessitates a personalized and multi-pronged approach Table 1:

**Table 1:** Comprehensive approach to care for cancer survivors

Aspect	Element 1	Element 2	Element 3	Element 4	Element 5
Central Focus	Cancer Survivor	Heart Health	Long-term Wellness	Quality of Life	Survivorship Care
Medical Management	Routine Monitoring	Cardiotoxicity Management	Lifestyle Interventions	Medication Interactions	Personalized Treatment Plans
Lifestyle Modification	Diet & Nutrition	Physical Activity	Stress Reduction	Weight Management	Smoking Cessation
Support Systems	Educational Workshops	Community Support	Family Involvement	Regular Screenings	Healthcare Coordination

**Fig. 1:**
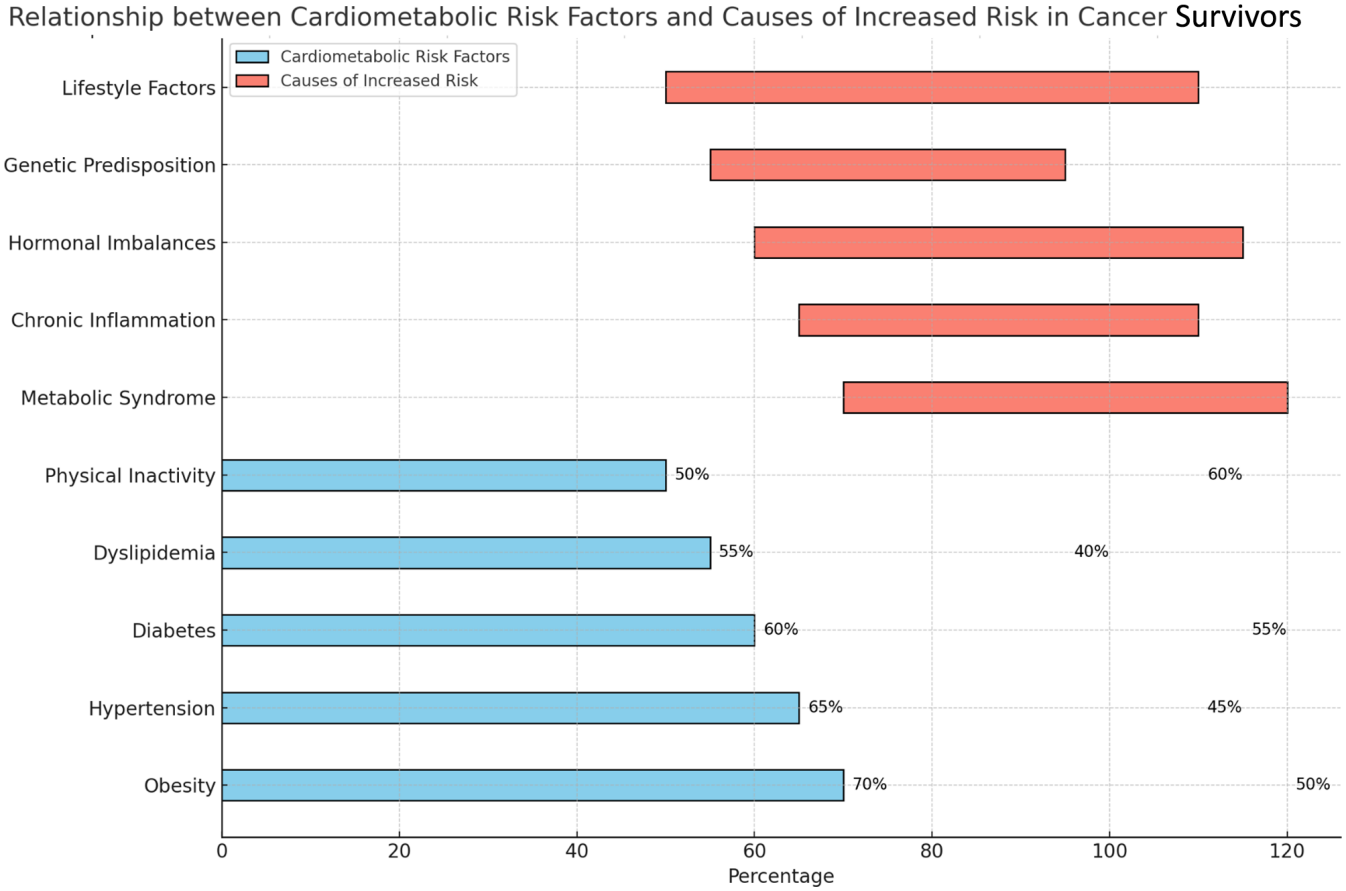
Cardiometabolic risk factors and chemo toxic effects in the cancer survivors

- Early identification and risk stratification: Prompt identification of at-risk individuals through screening and risk assessment is crucial for early intervention and tailored management [2]. Imaging data from cancer patients, such as the presence of atherosclerosis detected during oncologic workup, can predict and stratify cardiovascular outcomes. Factors such as pre-existing conditions, treatment type, and lifestyle habits inform individualized risk categorization.
- Dietary modifications: Promoting a balanced and healthy diet rich in fruits, vegetables, whole grains, and lean protein, while minimizing processed foods, unhealthy fats, and sugary drinks, plays a pivotal role in regulating blood pressure, lipids, and blood sugar levels [26]. Collaborating with registered dietitians can help survivors develop personalized meal plans to meet their specific needs and preferences [27].
- Physical activity: Regular physical activity, adapted to individual fitness levels and limitations, is essential for improving cardiovascular health, managing weight, and enhancing insulin sensitivity [28]. Exercise programs should be designed based on survivor preferences and physical abilities, gradually increasing intensity and duration over time [29].
- Medication management: Optimizing medication regimens while minimizing unnecessary polypharmacy plays a crucial role in managing cardiometabolic risks. Particular attention should be given to interactions between chemotherapy agents and cardiovascular medications, such as statins. Regularly reviewing medications, assessing potential interactions, and adjusting dosages as needed are essential tasks Fig. 1.

- Psychological support: Addressing the emotional and psychological toll of cancer and its treatment is essential for optimizing adherence to healthy lifestyle modifications and managing stress, a prominent contributor to hypertension [30]. Cognitive-behavioral therapy, mindfulness practices, and support groups can be valuable tools for managing stress and promoting emotional well-being.

Policy and Healthcare System Implications.

- Equitable healthcare access is crucial for managing cardiometabolic risks in cancer survivors, with disparities often influenced by socioeconomic status and insurance coverage [31].
- Policies should guarantee consistent access to comprehensive screenings and multidisciplinary care, including oncology, cardiology, and primary care.
- Guidelines, such as the 2022 ESC Cardio-Oncology guideline document, recommend regular monitoring and tailored management of cardiometabolic health in cancer survivors [32].
- Personalized care approaches and lifestyle modification support are essential to address individual risk factors and improve cardiometabolic outcomes for cancer survivors [2].

### Collaborative care

The complex nature of managing cardiometabolic risks in cancer survivors demands a collaborative approach involving a multidisciplinary team (Central illustration):

- Oncologists: Play a central role in coordinating care, monitoring treatment-related side effects, and adjusting treatment plans to minimize cardiometabolic risks.
- Cardiologists: Provide expertise in managing hypertension, dyslipidemia, and other cardiovascular conditions, collaborating with oncologists on medication regimens and treatment strategies.
- Primary care physicians: Serve as the cornerstone of long-term care, monitoring cardiometabolic risk factors, promoting healthy lifestyle modifications, and coordinating with specialists.
- Nutritionists and dietitians: Develop personalized meal plans and provide dietary guidance to support healthy eating habits and manage weight.
- Exercise physiologists: Design and supervise safe and effective exercise programs tailored to individual survivor needs and capabilities.
- Psychologists and mental health professionals: Offer support and interventions to address stress, anxiety, and depression, which can impede adherence to treatment and management strategies.

### Future directions

Despite significant advancements, several knowledge gaps remain:

- Long-term impact of specific therapies: Investigating the long-term cardiometabolic consequences of different cancer therapies is crucial for informing treatment decisions and developing personalized risk management strategies [33].
- Emerging risks: Further research is needed to understand and address the growing burden of obesity and metabolic syndrome in cancer survivors and develop effective interventions for prevention and management [23].
- Medication adherence: Strategies to improve adherence to lifestyle modifications and medication regimens are essential for optimizing cardiometabolic health in this population [34].
- Survivorship care models: Developing and evaluating comprehensive survivorship care models that integrate cardiometabolic risk management into routine cancer care are critical for ensuring long-term well-being [35].
- Longitudinal Studies: Emphasize the need for long-term studies to understand the evolving nature of cardiometabolic risks post-cancer treatment [36].
- Innovative Therapies: Explore potential advancements in treatment options that could minimize these risks.

## Conclusion

Cancer survivors are at a heightened risk for cardiovascular disease, which persists and evolves over the trajectory of survivorship. This augmented risk underscores the necessity for a collaborative, proactive approach in healthcare delivery, integrating cardiovascular risk management with oncologic care. Efforts should be directed towards developing and implementing robust clinical guidelines that address the cardiometabolic health of cancer survivors, with an emphasis on individualized risk assessment, early intervention, and sustained management.

## Data Availability

No datasets were generated or analysed during the current study.
